# Narrative Review of the Complex Interaction between Pain and Trauma in Children: A Focus on Biological Memory, Preclinical Data, and Epigenetic Processes

**DOI:** 10.3390/children10071217

**Published:** 2023-07-13

**Authors:** Veronica Rivi, Giovanna Rigillo, Ylenia Toscano, Cristina Benatti, Johanna Maria Catharina Blom

**Affiliations:** 1 Department of Biomedical, Metabolic and Neural Sciences, University of Modena and Reggio Emilia, 41125 Modena, Italy; veronica.rivi@unimore.it (V.R.); giovanna.rigillo@unimore.it (G.R.); ylenia.toscano@unimore.it (Y.T.); cristina.benatti@unimore.it (C.B.); 2 Centre of Neuroscience and Neurotechnology, University of Modena and Reggio Emilia, 41125 Modena, Italy

**Keywords:** pain, trauma, biological memory, preclinical studies, epigenetics

## Abstract

The incidence and collective impact of early adverse experiences, trauma, and pain continue to increase. This underscores the urgent need for translational efforts between clinical and preclinical research to better understand the underlying mechanisms and develop effective therapeutic approaches. As our understanding of these issues improves from studies in children and adolescents, we can create more precise preclinical models and ultimately translate our findings back to clinical practice. A multidisciplinary approach is essential for addressing the complex and wide-ranging effects of these experiences on individuals and society. This narrative review aims to (1) define pain and trauma experiences in childhood and adolescents, (2) discuss the relationship between pain and trauma, (3) consider the role of biological memory, (4) decipher the relationship between pain and trauma using preclinical data, and (5) examine the role of the environment by introducing the importance of epigenetic processes. The ultimate scope is to better understand the wide-ranging effects of trauma, abuse, and chronic pain on children and adolescents, how they occur, and how to prevent or mitigate their effects and develop effective treatment strategies that address both the underlying causes and the associated physiological and psychological effects.

## 1. Introduction

Children and adolescents who experience painful and/or traumatic events represent a vulnerable population with complex medical and psychosocial needs [1,2] that can affect their relationships, mood, behavior, and cognitive functions [3]. As childhood and early adolescence represent a period of active neuroplasticity, traumatic and/or painful events can induce long-lasting changes in the brain and nervous system, impairing neuroplasticity processes and hindering coping resources [4,5,6]. On the other hand, timely intervention during this (neuro)developmental phase may represent a successful strategy for mitigating the effects of such adverse events [7,8]. To further complicate this scenario, the effects of trauma and/or pain can be intergenerationally passed on via epigenetic mechanisms [8,9,10,11]. Given the vicious circle that exists between pain, trauma, memory, and epigenetics [1,2,12], a stronger dialogue between preclinical and clinical studies is both urgent and necessary [11,12,13,14].

As early-life trauma and pain, such as abuse and neglect, predict lifelong mental and physical health consequences [12,13,14,15,16,17], the identification of early biological, psychological, emotional, behavioral, and social changes induced by traumatic and/or painful experiences [18], allows researchers and clinicians to better understand their vulnerability and intervene in a proper and timely manner [19]. In other words, a comprehensive approach to address the requirements of children and adolescents with pain and/or trauma, will improve their long-term health outcomes and allow them to lead healthier and more fulfilling lives.

Given these premises, in this narrative review, we aim to give a comprehensive account of this topic, covering a range of specific questions using the PICO framework to formulate specific questions. We (1) focus on a specific population (i.e., children experiencing traumatic and/or painful events); (2) identify processes that modulate and are modulated via trauma and pain (i.e., biological memory and epigenetic mechanisms); and (3) propose a multidisciplinary and translational approach to explore how and what preclinical studies may contribute to deciphering the effects of painful and traumatic events during childhood.

**Figure 1:**
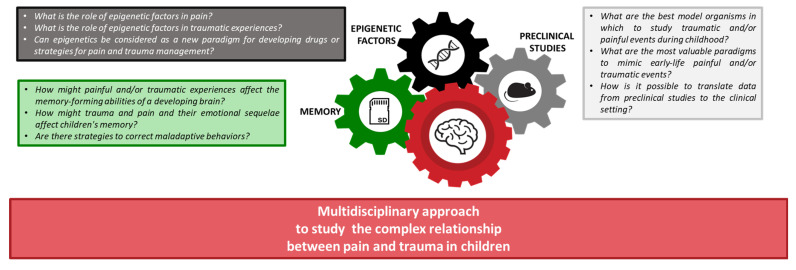
The framework of this narrative review aims at understanding the complex interaction between pain and trauma in children, focusing on biological memory, preclinical data, and epigenetic processes.

The key questions driving this narrative review are summarized below and reported in Figure 1:

- How might painful and/or traumatic experiences affect the memory-forming abilities of a developing brain?
- How might trauma and pain and their emotional sequelae affect children’s memory?
- Are there strategies to correct maladaptive behaviors?
- What is the role of epigenetic factors in pain?
- What is the role of epigenetic factors in traumatic experiences?
- Can epigenetics be considered a new paradigm for developing drugs or strategies for pain and trauma management?
- What are the best model organisms to study traumatic and/or painful events during childhood?
- What are the most valuable paradigms to mimic painful and/or traumatic early-life events?
- How can we translate data from preclinical studies to the clinical setting?

To answer these questions, we summarized the most recent and relevant scientific literature. In doing so, we aim to open new avenues for better understanding and studying the phenomenon of the relationship between pain and trauma in childhood. At the same time, we focus on how these three macro areas, biological memory, preclinical studies, and epigenetics, contribute to and are simultaneously modified via traumatic and/or painful experiences.

## 2. Methods

The literature research was conducted by researchers with diverse backgrounds and expertise in the fields of neuroscience, psychology, molecular biology, genetics, and neuropsychopharmacology. PubMed, PsycINFO, Web of Science, and the Cochrane Library were searched from 2013 to March 2023. Additionally, a manual search of the literature was performed, and the reference lists of the retrieved articles were examined. We only considered studies that adequately described and assessed pain and/or trauma in clinical or non-clinical populations of children (from 0 to 10 years old) and adolescents (from 10 to 19 years old). A total of 254 original investigations were included.

These studies encompassed research conducted on the general population, as well as clinical studies exploring the consequences of allostatic load/overload on both physical and mental health in various settings. The keywords used for the literature research include ‘pain in childhood’, ‘trauma in childhood’, ‘biological memory and pain’, ‘biological memory and trauma’, ‘early life stress models’, ‘preclinical studies’, ‘stress and epigenetics’, ‘epigenetics and trauma’, ‘epigenetics and pain’, and ‘epigenetics and adverse childhood experiences’.

## 3. The Double-Edged Sword in Defining the Relationship between Pain and Trauma

### 3.1. Definition of Trauma

According to the Substance Abuse and Mental Health Services Administration, trauma ‘results from an event, series of events, or set of circumstances that are experienced by an individual as physically or emotionally harmful or threatening and that has lasting adverse effects on the individual’s functioning and physical, social, emotional, or spiritual well-being’ [1,2]. The definition of trauma has evolved and expanded over the past four decades as knowledge regarding life experiences that cause psychological distress has grown [20,21]. Alongside this, the field has repeatedly tackled what exactly constitutes trauma. What is known is that trauma is a pervasive problem that results from exposure to an incident or series of events that are emotionally disturbing or life-threatening with lasting adverse effects on the individual’s functioning and mental, physical, social, emotional, and/or spiritual wellbeing [21].

Physical trauma includes blunt force and internal trauma [22], such as those caused by sexual abuse, transportation accidents, invasive medical procedures, natural disasters, domestic or community violence, assault, and terrorism [23].

Emotional trauma, instead, mainly affects key brain areas for emotions, emotional behavior, and motivation, including the amygdala, hippocampus, and prefrontal cortex, and occurs after distressing events, inducing psychiatric symptoms (i.e., negative thoughts, denial, anxiety, and panic attacks) as well as attention and learning deficits [24].

Emotional trauma can be acute, chronic, or complex. Acute trauma is typically related to a single event, lasts no longer than four weeks, and occurs within the first three days following the event [25]. Its symptoms include distress triggered by reminders of the event, avoidance behaviors, difficulty in sleeping, repetitive nightmares, amnesia about the event, and higher reflexes [26].

Chronic trauma is related to ongoing trauma that arises when distressing events are experienced on several occasions [27], whereas complex trauma refers to the experience of chronic trauma with long-term emotional and physical symptoms [24].

Given their severe and pervasive nature, traumatic events can disrupt many aspects of children’s neurodevelopment, impairing cognitive performances and social relations, causing disruptive behaviors, and inducing anxiety and depression, as well as excessive arousal [26]. Chronically traumatized children are often unable to self-regulate and, therefore, experience intense feelings without the ability to identify and communicate them [24]. These problems may extend from childhood to adolescence and adulthood [26].

Furthermore, chronic trauma in childhood, by lowering the threshold for re-traumatization, places children at risk for additional traumatic experiences, leading to higher risks for psychiatric and addictive disorders [28,29].

### 3.2. Definition of Pain

Pain can be defined as an ‘unpleasant experience associated with, or resembling that associated with, actual or potential tissue damage’ [30]. While pain plays a key role in avoiding physical harm, if unrelieved, it may be psychologically and physiologically harmful. Painful experiences are processed and modulated by several neuronal circuits, involving the brainstem, thalamus, cortical subplate, and cortex [31]. While the brain areas involved in pain perception are completely developed by 24 weeks of gestation, the descending inhibitory circuits that control pain are still immature at birth [31,32]. Therefore, neonates are likely to be more sensitive to pain than older children and adults [31,32]. Although every child may experience pain from the earliest stages of life, childhood pain is one of the most misunderstood, underdiagnosed, and under-treated medical problems [33,34].

Epidemiological studies revealed that more than one-third of children suffer from abdominal pain [35]; nearly 250 thousand children younger than 16 years old suffer from juvenile arthritis [36]; 20% of children aged 5–17 years suffer headaches [37]; and of 1.5 million children who received surgery, 20% receive inadequate pain relief [38,39]. However, unaddressed pain may be stressful and even traumatizing, leading, therefore, to higher pain perception [40].

Importantly, each child has a different pain perception and attributes to pain, i.e., a personal ‘meaning’ [41]. Thus, developing valid and reproducible clinical approaches and a shared glossary to assess a child’s concept of pain is necessary for preventing childhood pain and its consequences. In particular, as reported by Maitre et al. (2017), early painful experiences can shape the somatosensory scaffolding of later cognitive, perceptual, and social development [42,43]. This risk is even higher in premature children [43]. For example, reduced amygdala and thalamus volumes and poor cognitive outcomes (i.e., language and attention deficits, poor visual motor functions, lower IQ score, and poor behavioral outcomes) have been observed in children born very preterm and subjected to invasive procedures [44,45,46].

Pain can be classified into three broad categories: nociceptive, neuropathic, and nociplastic [47]. Nociceptive pain derives from tissue injury and is usually well localized and precisely described by patients [48], whereas neuropathic pain occurs with injury or insult to a peripheral or central nerve [49].

Finally, nociplastic pain is characterized by changes in nociception, even though there is no clear indication of actual or potential tissue damage that would typically activate peripheral nociceptors. Similarly, no evidence of any disease or injury to the somatosensory system would explain the presence of pain [30,50,51].

Pain can be both acute and chronic. As reported by the IASP (International Association for the Study of Pain), acute pain is caused by a specific disease or injury, has a useful biologic meaning, and is self-limited, chronic pain persists or recurs for longer than 3 months, can continue even if the injury has been treated, serves no biological purpose, may arise from psychological states, and has no recognizable end-points (IASP, 2019) [52]. Thus, the therapeutic interventions for acute and chronic pain are different: for treating acute pain, it is necessary to interrupt the nociceptive signals, whereas the therapy of chronic pain relies on multidisciplinary pharmacological and non-pharmacological approaches and personalized treatments [52].

Acute pain can progress into chronic pain via several pathophysiological and histopathological steps that lead to impaired central sensitization, causing allodynia (i.e., pain elicited via a stimulus that does not usually provoke pain) and hyperalgesia (increased pain from a stimulus that usually provokes pain) [53]. Theoretically, if the pathophysiological changes during this transition can be prevented or reversed, it is possible to prevent or minimize chronic pain and its dramatic effects. To date, numerous studies attempted to develop valid strategies to prevent this transition, but so far, there has been very limited promising evidence. Therefore, researchers are called to identify risk factors that allow for the early recognition of patients with a high risk of developing chronic pain. This is imperative in reducing chronic pain development, especially in children.

### 3.3. Pain, Trauma, and Their Complex and Multifaced Relationship

The relationship between trauma and pain in children is complex, multifaceted, and less well-documented than in adults [54]. Research shows that traumatic experiences can significantly impact a child’s perception and experience of pain and profoundly interfere with a child’s overall development, including their ability to regulate and process pain. Trauma frequently persists even after the traumatic event has ended and often heightens a child’s sensitivity to pain, leading to a lowered pain threshold [55]. Cohen et al. (2017) found that trauma-exposed children often express their emotional distress through physical complaints and somatic symptoms [56], such as headaches, stomachaches, muscle aches, or other unexplained bodily discomforts often associated with stress and trauma [57]. Thus, trauma and pain have a reciprocal relationship, influencing and exacerbating each other.

Physical pain can trigger traumatic memories or emotions, leading to re-traumatization and a worsening of psychological distress [55,58]. Similarly, trauma-related stress and psychological factors can amplify the experience of physical pain. Also, trauma can disrupt a child’s ability to effectively cope with pain [59]. They may develop maladaptive coping strategies or engage in avoidance behaviors to deal with the pain, further perpetuating their distress and hindering their recovery [55,58]. Finally, unresolved trauma in childhood may have long-lasting effects on pain perception and pain management and contribute to the development of chronic pain conditions, such as fibromyalgia or complex regional pain syndrome, but also increase the risk of post-traumatic stress and enhance the risk of experiencing pain-related disabilities later in life [60]. Together, these highlight the complex interplay between trauma and pain, where emotional distress can manifest as physical pain and physical pain can trigger traumatic memories or emotions [61,62,63]. Understanding and addressing the relationship between trauma and pain, then, is crucial for providing appropriate care and support to traumatized children as well as to children experiencing pain [55,56,57,61].

Given these premises, in the next paragraphs, we will explore three important realms to gain a better understanding and allow us to recognize and address the relationship between trauma and pain in children more effectively. The three areas we will explore are the impact of biological memory, the critical role of the environment, and the use of models to improve our neurobiological and molecular insight [59,64,65]. A comprehensive approach that integrates trauma-informed care [58], appropriate psychological support, and pain management strategies will alleviate the impact of trauma on pain experiences and promote the child’s overall well-being.

## 4. Pain, Trauma, and Biological Memory: A Complex Trinomen

Memory biases for previous painful and/or traumatic experiences are known to be strong predictors of adolescents and adult suffering from psychiatric disorders [66]. Trauma-exposed individuals (regardless of clinical presentation) often tend to retrieve memories of events from their past in a generalized way, with a lack of event-specific details [67]. These impaired memories have been implicated in the development and maintenance of psychiatric disorders, such as post-traumatic stress disorder (PTSD) and major depressive disorder [30,31,32]. Furthermore, memory biases for previous pain experiences are known to be strong predictors of post-surgical pain outcomes in children [68].

Additionally, a recent study conducted over a period of time found that adults who had fewer specific memories of pain before undergoing major surgery were more likely to develop chronic post-surgical pain even up to 12 months later [33]. Nonetheless, most of the studies investigating pain memories in children so far have primarily relied on comparing responses from single-item pain assessments administered during the painful event and again during recall.

While this approach captures the sensory and emotional aspects of memory, it fails to evaluate the spatiotemporal and perceptual qualities that are essential for autobiographical memory [69,70,71]. The lack of research in pediatric populations highlights the urgent need for a comprehensive investigation. Thus, there is a crucial need for a broader understanding of autobiographical memory and exploring strategies that can advance our assessment and methodologies and enhance our understanding of children’s memory of traumatic and/or painful experiences. Such advancements have implications for developing targeted interventions to prevent and/or manage pain and trauma by enhancing specific aspects of memory [33].

### 4.1. How Painful and/or Traumatic Experiences May Affect the Memory-Forming Abilities of a Developing Brain?

Our memories define our sense of self, drive our decisions and thoughts, affect our emotions, and allow us to adapt to ever-changing environments [72]. Since our first breath, our physical growth is accompanied by memory development [73], which, in turn, is necessary for the formation of complex cognitive abilities, including language, problem-solving, and the overall ability to form a sense of self [74].

During the first months of life, new behaviors are learned via imitation, whereas memory is expressed and measured via changes in behaviors [75]. This type of memory is called non-declarative (or procedural or implicit) memory. As children get older, they develop declarative (or explicit) memory, which allows them to remember facts and experiences [76]. These processes are associated with the development of autobiographical memory, a uniquely human form of memory that goes beyond the recall of experienced events and integrates perception, interpretation, and evaluation across the self, others, and time to create a personal history [77]. The ability to accurately remember previous events is of vital importance in the anticipation of, and response to, future events, especially if they are potentially painful, stressful, and/or traumatizing [78].

On the other hand, traumatic and/or painful experiences during a period of heightened brain plasticity like childhood often permanently interfere with the development of the functional brain circuits involved in memory formation and the behavioral responses to environmental stimuli [79,80]. Many studies demonstrated that traumatic and/or painful experiences during childhood may lead to dramatic alterations in memory functioning, especially autobiographical memory [67]. For example, infants and children who have been abused, neglected, or exposed to multiple medical and painful procedures often exhibit profound impairments in cognition, including intellectual developmental delays and language and psychomotor deficiencies, and have a higher risk for poor academic achievement [81,82,83]. Furthermore, pain and trauma in childhood might increase vulnerability in other stressful situations occurring in adolescence and adulthood, leading to the development of a broad range of psychiatric disorders [84,85], which, in turn, are associated with cognitive deficits and memory impairment [86,87,88,89]. Thus, although trauma and pain in childhood represent a serious psychosocial, medical, and public policy problem, their effects on the memory of children are still debated and under investigation [90,91].

Given the complex relationship between pain, trauma, and memory, disagreement in the literature is not too surprising. While some experts believe that trauma and pain can lead to memory fragmentation or disorganization [92,93,94], others propose the dissociation of trauma memories from other autobiographical memories [94,95,96,97]. 

Furthermore, certain researchers suggest that traumatic and/or painful events not only lead to widespread changes in memory functioning but are also responsible for increased susceptibility to memory distortion or impaired memory retrieval of specific autobiographical memories [98,99,100,101,102,103].

Overall, growing evidence suggests that memories of traumatic or painful autobiographical experiences are unconsciously blocked for many years and recovered later, especially after psychiatric therapy [104]. Repressed memories are believed to act as an automatic and unconscious defense mechanism to protect people who experienced a traumatic event, making them unaware of having been abused or traumatized [105]. At the same time, therapeutic interventions may lead to the creation of false memories of negative events in children [101]. For example, Otgaar et al. (2008) show that in children, a negative false event (e.g., being accused of copying) is more easily implanted than a neutral false event (e.g., moving to another classroom) [106,107]. Finally, several studies indicate that traumatic experiences are generally well remembered [108,109,110]. Importantly, individual differences in trauma and/or pain history and psychopathology may differentially affect the accuracy of long-term memory for stressful life events [111]. Furthermore, gender differences in memory for emotional childhood events emerged, with males remembering fewer emotional childhood experiences compared to females [112]. Thus, the relationship between memory, trauma, and painful experiences is a complex problem both at the clinical as well as legal level [113] and certainly cannot be generalized, especially when it comes to children, who are more vulnerable to the formation of false memories.

### 4.2. How Might Trauma and Pain and Their Emotional Sequelae Affect Children’s Memory?

Childhood trauma and pain alter affective and neurobiological development and place individuals at increased risks of learning and memory deficits, relational problems with peers, and psychiatric disorders [114], including anxiety, depression, PTSD, substance abuse, and disruptive behaviors later in their lives [115,116,117,118,119,120]. As adults, these children continue to be at higher risks for psychiatric disorders, drug and alcohol abuse, serious medical illnesses, and lower economic productivity [117]. Thus, identifying mechanisms that underlie the strong link between childhood trauma and psychopathology, as well as factors that may protect against this risk, is of vital importance to develop targets for preventive interventions.

A central mechanism involved in the etiology of trauma-related psychopathology is the disruption of learning processes involved in the acquisition and extinction of conditioned fear. However, this is rarely studied in children [121]. For example, McLaughlin and coworkers (2016) demonstrate that childhood maltreatment is associated with the failure to discriminate between threat and safety cues during fear conditioning, reflecting either an enhanced fear generalization or a deficit in associative learning [116]. These results are important as atypical fear conditioning has been observed in multiple forms of psychopathology, including PTSD, which is characterized by a failure to extinguish fear memory [122], and anxiety disorders, which are associated with exaggerated fear responses during conditioning and extinction learning [123]. Furthermore, PTSD is frequently co-morbid with depressive and anxiety disorders [124], both of which are associated with memory impairment and trouble concentrating [125]. Eisen and colleagues (2007) investigated memory and suggestibility in the context of ongoing child maltreatment investigations [13] and found that maltreated children were more physiologically distressed during medical procedures and showed higher memory accuracy for the negative experience than non-maltreated children [13]. These results not only underline the strong and reciprocal relationship between trauma and pain but also suggest that in children with a history of maltreatment, stressful situations activate dissociative defense strategies, which, in turn, affect information processing. However, not all children who experience severe pain and/or trauma suffer these negative consequences. The outcomes of painful and traumatic experiences in children can vary greatly depending on factors such as the timing of the abuse or neglect during the child’s development, the chronicity of the experiences, and their duration and frequency. These variables can result in a wide range of outcomes, highlighting the diverse and individualized impact of such experiences on children [117].

### 4.3. Strategies to Correct Maladaptive Behaviors: From Retrieval-Induced Forgetting to Enriched Environments

Trauma survivors often show intrusive recall and an altered capacity to intentionally forget [126]. Forgetting results from active processes aimed at suppressing the retrieval of unwanted memories. Thus, deficits in forgetting memories of traumatic/painful experiences may play a key role in the onset of psychopathological disorders [127]. However, interventions aimed at suppressing disturbing autobiographical memories have proven to be useful for the prevention and treatment of complex trauma, such as PTSD [128]. One of these interventions is the directed forgetting paradigm adopted for patients suffering from PTSD, where participants are required to forget experimental items that have just been presented. Although these patients show great difficulties in performing this task, recent studies demonstrate that when they are taught to associate aversive scenes with naturalistic reminders and then to practice voluntarily suppressing the scenes when cued with the reminders, the inhibitory control of memory retrieval can be assessed [96].

Another approach widely used is retrieval-induced forgetting, which is an explicit unintentional form of forgetting whereby the selective retrieval of trauma-related stimuli leads to the enhancement of induced forgetting for individuals with traumatic and/or painful early-life experiences [129]. Thus, even if retrieval suppression is severely compromised in traumatized patients showing deficits in suppression-induced forgetting, these procedures and their promising results raise the possibility that therapeutic approaches that attempt to have patients confront and then suppress their traumatic memories might be a valuable addition to standard psychological treatments [96]. In this context, clinicians working with children or adults who have experienced trauma are called to create a therapeutic environment that supports the recollection and psychological processing of the impact and meaning of that trauma, not only the therapeutic environment but also the context in which these patients live may be beneficial for their recovery [130]. 

Research on brain plasticity suggests that the environment can shape brain structure and function and improve neuroplasticity and the effects of the treatment of psychiatric disorders [130].

In this context, preclinical studies are of fundamental importance for investigating the conserved molecular and neurological mechanisms through which pain, stress, and trauma are involved in the etiopathogenesis of psychiatric disorders and the role of both genes and the environment regarding their progression or recovery.

## 5. Preclinical Studies to Decipher the Relationship between Pain and Trauma

Over the last decade, great scientific effort has been devoted to unraveling the neural and molecular mechanisms underlying pain and trauma, paving the way to novel or improved treatment approaches for trauma and pain-related disorders [131]. In this context, animal models of human diseases represent major scientific tools [132], allowing extremely fine characterizations of the molecular, neuronal, and anatomical mechanisms underlying pain and trauma and the standardization of genetic and environmental backgrounds [133]. Valid animal models should possess similarities in symptoms, reproduce the neurobiological bases of the human condition, and predict the responsiveness to drugs currently used to treat the disorder [134].

### 5.1. Importance of Pre-Clinical Studies to Investigate the Conserved Molecular, Cellular, and Behavioral Effects of Pain

Pain is a subjective phenomenon; therefore, in both animal models and humans, it can be studied via behavioral tests [135]. The unpleasant emotional experience of pain in animal models is deduced from pain-like behaviors, like reduced ambulation, agitation, increased grooming of the affected area, and vocalizations upon sensory stimulation [135,136,137]. The most commonly used method to quantify nociception in animal studies is the withdrawal from a nociceptive stimulus: withdrawal from a stimulus that does not normally evoke it is considered allodynia, whereas withdrawing with an exaggerated response indicates hyperalgesia [136]. However, the results of these tests can be influenced by the subjectivity of the investigators who determine if the withdrawal represents a pain response or whether it is due to other factors (e.g., tickle, grooming, or ambulation). Therefore, depending on the cut-off points used by researchers, what constitutes pain-related behavior can significantly vary between laboratories. Furthermore, no single behavioral test can capture the full spectrum of nociception in non-communicating subjects [91,92]. In fact, unlike other mammals, humans can describe their feelings via speech, which provides quick and valid introspective ratings and descriptors, especially in the case of chronic pain, which often has no obvious cause [138] and can be associated with complex autobiographical content that is be easily captured in animal experiments [139].

As reported in the first section of this narrative review, pain is a multifaceted and diverse experience that can be categorized into several types and modalities, depending on the presentation and triggering stimulus of the pain event. Thus, the advantages and disadvantages of each model and behavioral test should be taken into account to obtain objective and meaningful results that will improve our understanding and management of pain [140]. A great example of success in pain translational research is the development of anti-calcitonin gene-related peptide antibodies, which, after 30 years of preclinical studies, have been approved for the prevention and treatment of migraines [141]. One of the main goals of pain research is the characterization of the conserved physiological and molecular mechanisms underlying pain, especially chronic pain, as treatments remain suboptimal. This goal cannot be reached without the involvement of animal models. However, to lead to far more effective and translatable (“bedside-to-bench-to-bedside”) pain research, preclinical studies should be combined with clinical ones, including, for example, human imaging.

### 5.2. Importance of Pre-Clinical Studies to Investigate the Conserved Molecular, Cellular, and Behavioral Effects of Trauma

Numerous animal models have proven to be valid tools for studying the effects of trauma and stress exposure within experimental paradigms. For example, it is possible to control the specific type of traumatic event (e.g., forced swimming or social deprivation), the duration of exposure (e.g., acute vs. chronic), and the developmental period of exposure. However, many preclinical studies regarding traumatic events have been performed in controlled environmental and genetic conditions [142]. Therefore, for the models to be truly representative of human beings and ecologically valid, researchers must carefully select the individual differences (e.g., gender), choose the appropriate risk factors and types of trauma, and define the behavioral measures of symptoms.

### 5.3. Early-Life Stress and Consequences of Trauma and Pain-Related Disorders

Traumatic and/or painful experiences during childhood and adolescence may function as a neurodevelopmental assault that, for example, increases the likelihood of developing psychosis [131]. Thus, preclinical studies are of fundamental importance for deciphering the mechanisms underlying the effects of early traumatic, painful, stressful experiences on the risk for psychiatric disorders in adulthood. 

‘Childhood’ in rodents corresponds to the time from post-natal day (PND) 8 to PND 20, whereas the juvenile age starts from PND 21 [143]. Stressful, traumatic, or painful experiences during these stages not only have detrimental effects on brain neuroplasticity and functioning but are also associated with a higher risk of mental illness in adulthood [144]. To study how negative early-life events predispose children to psychiatric disorders and behavioral dysfunction in adulthood, researchers usually focus on changes in cognitive ability, which represent a core component of psychosis [145,146,147,148]. 

In particular, cognitive deficits commonly observed in psychiatric disorders like schizophrenia, depression, and PTSD that can be measured in animal models include working memory deficits and impaired cognitive flexibility [149,150,151]. Preclinical and clinical evidence indicates that early-life stress (ELS) dysregulates the hypothalamic–pituitary–adrenal (HPA) axis and autonomic nervous system (ANS), which are involved in the re-establishment and maintenance of homeostasis after traumatic, painful, and stressful events [152]. The respective contributions of the neuroendocrine and autonomic systems depend on the type of stressors, their intensity, and how they are perceived by the organism [153]. The HPA axis response to stress can be considered a mirror of the organism’s response to stress. While the response to acute stress is generally considered adaptive, excessive or prolonged stress can lead to deleterious effects [154], especially on cognitive functions and the formation of memory, which, as described in the previous sections, play a key role in the trauma–pain–neurodevelopment circuit.

Furthermore, the limbic regions, responsible for regulating the stress response, are also importantly involved in memory formation. Thus, together, these areas allow organisms to tailor their responses to acute stressors based on their prior experience and anticipated outcomes [144,155,156,157].

However, chronic stress alters the neuroplasticity of the central stress-processing network, including the HPA axis and ANS. Therefore, stressful early-life events like painful and/or traumatic experiences induce persistent changes in the ability of the HPA axis and ANS to respond to stress in adulthood, increasing, therefore, the risk for psychiatric conditions, cognitive dysfunction, and memory impairment [156]. Given the high level of conservation of the stress response between animal models and humans [158], preclinical studies are of fundamental importance for further investigating the long-lasting consequences of ELS on cognitive function and the risk of developing stress-related psychopathology later in adulthood.

#### Maternal Separation as an Example of ELS

Frequent and prolonged contact between the mother and her pups is necessary for offspring survival and healthy development [159]. During the lactation period, the mother provides food, protects her pups, and teaches them how to survive and how to relate to their other littermates [160]. Together with licking, grooming, arched-back nursing, and building the nest, these phenomena belong to what is called maternal care [161]. There is a wide variety of early-life stress protocols that use these behaviors, but studies differ greatly regarding the separation period and duration, temperature regulation, and pup isolation (see [10,16] for a critical review of the behavioral procedure).

Despite the naturally occurring variability in maternal care, preclinical studies demonstrate that tampering with the interaction between the mother and her pups, both in the neonatal period (PND 0-7) or during childhood (PND 8-20), can generate a stressful condition early in life [162]. Pups raised by a mother exhibiting low levels of maternal care display neurobiological alteration (e.g., structural, functional, biochemical, and molecular), changed HPA axis functioning [163], anxiety and depressive-like behaviors, and higher levels of cocaine/alcohol consumption, resembling the phenotypes found in humans exposed to poor maternal caregiving [164].

### 5.4. What Are the Possible Barriers That May Limit Preclinical Studies?

Although no animal model can recapitulate the complex mechanisms through which traumatic and/or painful early-life events may alter neurodevelopment and cognitive functions, these organisms provided a great contribution to our understanding of the neurobiology of psychiatric disorders. To date, results of preclinical studies have been mixed, with some research suggesting that ELS promotes anxiety-like behaviors and/or increases the susceptibility to subsequent stressors. Other studies suggest that ELS reduces anxiety-like behavior and/or confers resilience to subsequent stress exposure [124,126,127,144,145,146,147,148]. Factors such as sex and the timing and severity of early-life and adult stress exposure seem to play a key role in determining whether a particular early-life experience would promote adaptive or maladaptive behavior later in life. Thus, researchers are now called to pay attention to individual differences that may impact different risk factors, the type of trauma, and the behavioral measures of symptoms before drawing conclusions [165,166,167,168,169]. These aspects are critical to be able to relate behavioral symptoms with their neural correlates and to establish effective drug development and drug testing platforms. We understand that many people object to animal research, especially animal pain, and trauma research, on principle. However, our aim here is to summarize the results obtained using these models and emphasize their important contribution to deciphering the conserved mechanisms through which childhood pain and trauma may affect neurodevelopment.

## 6. Epigenetics of Childhood Trauma and Pain

Studies investigating variations in maternal care in rodents provide the first evidence that epigenetic factors (i.e., DNA methylation, histone modifications, and small non-coding RNAs) may have a critical role in the developing brain [170]. These studies paved the way for a new research area aimed at exploring how the developmental effects of traumatic and/or painful experiences lead to lifelong changes in neurobiology and behavior. Thus, epigenetics is an evolving research field that has progressed rapidly in the past decade, fueled by advances in molecular biology and brain imaging that highly contributed to a better understanding of the biological impact of a broad range of environmental experiences, which include trauma and pain and may influence subsequent generations of offspring [8].

### 6.1. Epigenetics and Pain: New Insights about an Old Problem

One of the most accredited hypotheses to explain the difference in pain perception, modulation, and physiology are genetic modifications associated with the pain response. The alteration of gene expression causes structural and functional modifications in neural circuits and plasticity. Indeed, biological mechanisms such as genetic modifications contribute to the pain response, its duration, and intensity. Gene expression is under the direct control of transcription factors but it may also be influenced by epigenetic modulators.

The mechanisms of epigenetic regulation are of particular interest in the study of the interaction between genetic backgrounds and environmental conditions because they provide insight into how external stimuli may affect gene expression and add an important level of regulation to gene transcription. Epigenetic processes, comprising DNA methylation, histone modifications, and non-coding RNA (ncRNA), are receiving increasing attention within the pain research field [171,172,173]. Epigenetic modifications are hypothesized to mediate the transition from acute to chronic pain by gradually and progressively converting the injury experience into a pathological process of neuroinflammation, central sensitization, and ultimately chronic pain. Injury-induced changes in chromatin structure drive changes in gene expression and neuronal activity.

Preclinical studies suggest that chronic pain development is correlated to changes in gene expression resulting in crucial long-term alterations in neural activity [16,171,172]. Emerging evidence reports how epigenetics may modify general types of pain, including inflammatory, neuropathic, visceral, and cancer-related pain, via the alteration in chromatin structure [16]. Moreover, stressful stimuli trigger epigenetic changes in sensitive brain regions and in the immune and endocrine systems determining a dysregulation and an altered response [131]. Research on epigenetic mechanisms not only aims to understand the molecular and cellular processes involved in the pain response but has also received interest in the clinical community as potential new targets for the management of chronic pain [132]. 

#### 6.1.1. Understanding Pain Perception or Distortion from an Epigenetics Point of View

Accumulating evidence has demonstrated that epigenetic mechanisms represent an important level of the control of gene expressions in neural perception, modulation, and sensitization at both the central and peripheral levels and contribute to long-term plasticity in the central nervous system (CNS) [16]. The alteration of the transcriptional profile, a consequence of epigenetic modulation, entails a cell maladaptive change in the pain condition at the cellular level, in particular, changes that involve the main pain-related anatomical sites. The first is represented by peripheral sensory neurons, responsible for the detection of noxious stimuli, and transmission of nociceptive inputs from the periphery, via the dorsal root ganglia (DRG), to the spinal cord in the CNS. The second region of pain processing is represented by the dorsal horn of the spinal cord [153].

From a mechanistic point of view, epigenetic modifications control pain-related gene transcription differently as follows:

1. By modulating the expression of ion channels leading to changes in the excitability of neural circuits;
2. By inducing or repressing postsynaptic receptors and signaling molecules;
3. By altering cell numbers involved in pain transmission circuits, thus affecting their responsiveness;
4. By maladapting the structural plasticity at the pre- or post-synaptic level [16].

#### 6.1.2. DNA Methylation in Pain

DNA methylation is a biological process that plays an important role in pain mechanisms involving various brain structures and is supported by evidence suggesting the crucial function of methylation in persistent pain. Indeed, beyond the alteration in the global methylation of DNA, many studies highlight an increase in the methylation state of chronic pain-related promoter genes [174]. The modification consists of the addition of methyl group to the fifth carbon of cytosine residues situated adjacent to a guanine residue (CpG site), generally highly concentrated at the level of the promoter region of genes. The level of DNA methylation is guaranteed and mediated via the enzyme family of methyltransferases (DNMTs) that includes DNMT1, DNMT3a, and DNMT3b, and demethylation enzymes. Generally, the methylation of gene promoters is associated with the repression of gene transcription depending on the target residues and is the result of different types of interference, physically with the binding of transcription factors or serving as docking sites for methyl-CpG-binding domain proteins (MBDs), including MBD1-4 and MeCP2.

Also, the DNA methylation status is critical for the development and/or maintenance of hypersensitivity to pain, persistently or reversibly. Many studies associate an increase in the methylation state in the promoter of pain-related genes, such as *Oprm1* (encoding for the mu-opioid receptor MOR) and *Kcna2* (Potassium Voltage-Gated Channel Subfamily A Member 2), with a consequent reduction in their protein levels [175,176]. Nerve injury-induced changes in DNA methylation correlate with spatial and functional implications depending on the pain conditions. 

Furthermore, preclinical studies have identified an increase in the methylation state of the MOR and kappa opioid receptors in DRG neurons following nerve injury, suggesting a potential mechanism of opioid resistance in neuropathic pain [176,177,178].

In a mouse model of neuropathic pain, contrasting observations have been made regarding the methylation states in gene promoter regions. Specifically, hypomethylation has been detected in the promoter region of chemokine receptor 3 (CXCR3), which plays a crucial role in the inflammatory response process. Additionally, hypomethylation has also been observed in the gene G protein-coupled Receptor 51 (GPR151), which is implicated in DRG hyperexcitability [179,180]. Methylation changes have been reported in different forms of chronic pain, including chronic inflammatory or visceral pain, fibromyalgia, and rheumatoid arthritis [181]. In particular, peripheral inflammation induces the DNA methylation of the nerve growth factor (NGF) [178,182] or cystathionine-β-synthase (CBS) [183] gene promoter leading to increased pain. Moreover, in a rodent model of visceral pain, significant epigenetic regulation was observed. Specifically, an increase in methylation of the glucocorticoid receptor (GR) gene was reported in the context of a chronic water avoidance model. This increase in methylation was accompanied by a decrease in the expression of the GR gene [184].

#### 6.1.3. Histone Modifications in Pain

Histone modifications consist of post-translationally changes on the N-terminal histone tail, resulting in a remodeling of the chromatin structure and, thus, in gene expression. Among these modifications, histone acetylation, driven by histone acetyltransferase (HAT), enhances chromatin-promoting gene transcription. In contrast, histone deacetylation, catalyzed via histone deacetylase (HDAC), shapes condensed chromatin causing gene silencing [185,186]. Emerging evidence indicates that the enhanced acetylation of histones induces the transcription of pain-related genes in neuropathic pain [187]. Recently, there has been growing attention to the role of HDAC (histone deacetylase) activity in pain, leading to the development of various HDAC inhibitors aimed at promoting analgesia. However, it is worth noting that these inhibitors do not currently consider the diverse activation patterns within the entire HDAC subclass. Pharmacological studies indicate that HDAC inhibition improves hypersensitivity and alleviates nociception sensitization of both inflammatory and neuropathic pain by preventing adaptation in gene expression.

Indeed, the administration of HDAC inhibitors in a mouse model of chronic inflammatory pain has been shown to induce analgesia. This analgesic effect is achieved by upregulating mGluR2 receptors in the DRG [174]. Other evidence indicates that peripheral inflammation upregulates specific HDAC members leading to the reduction of histone acetylation in the spinal dorsal horn and spinal cord nerve [176,182,188]. In addition, it induces gene silencing resulting in the loss of pharmacological targets for analgesic therapy.

Histone modifications are also involved in the regulation and development of opioid tolerance and opioid-induced hyperalgesia in neuropathic pain conditions. In the context of nerve injury, it has been observed that there is an upregulation of HDAC, leading to a decrease in histone acetylation at the promoter level of pain-related genes such as opioid receptors (MOR), Kv4.3, and Nav1.8. Conversely, an increase in histone acetylation is observed at the promoter regions of BDNF and CdK5 in the dorsal root ganglia (DRG), contributing to the maintenance of neuropathic pain [16,189,190]. HAT inhibitors exert an antinociceptive effect by suppressing the hyperacetylation of histones at the promoter region of the gene encoding for macrophage inflammatory protein 2 (MIP-2), receptor chemokine CC motif receptor 2 (CXCR2), and cyclooxygenase-2 (COX-2) [191,192]. Moreover, HAT inhibition produces an analgesic effect in other persistent pain conditions, such as the incision model [177], and reduces the development of morphine-induced mechanical allodynia, thermal hyperalgesia, tolerance, and physical dependence [193,194]. 

While the exact role of histone methylation in the development of neuropathic pain still requires further clarification, there is evidence suggesting its involvement in driving gene expression within the dorsal DRG. This indicates that histone methylation plays a role in the molecular mechanisms underlying neuropathic pain [16].

Moreover, preclinical studies focusing on nerve injury models have revealed the significant role of histone methyltransferases, such as G9a, in pain modulation. These enzymes are involved in controlling the expression of genes such as Oprm1 (opioid receptor mu 1) and cannabinoid receptors CB1 and CB2 [19,187] and repressing dopamine levels [195,196,197]. Additionally, arginine histone methylation has also been associated with pain [198]. In terms of histone phosphorylation, it has been observed that certain early immediate genes, including c-fos, c-jun, and c-myc, are transcriptionally activated in response to external signals [16,196]. These findings highlight the involvement of histone modifications in pain-related mechanisms.

#### 6.1.4. Non-Coding RNA

The role of epigenetic modification has also been attributed to non-coding RNAs (ncRNAs), a cluster of RNAs that do not encode for a functional protein but can regulate gene expression at the post-transcriptional level, thus producing a biological effect. The ncRNAs include the long ncRNAs (lncRNAs), circular RNAs (circRNA), microRNAs (miRNAs), and naturally generated antisense RNAs (AS-RNAs), which regulate transcription, translation, and mRNA stability [181].

Growing research regarding the role of lncRNA in chronic pain has identified different lncRNAs performing different modes of action in pain models by interacting and interfering with miRNAs or directly targeting molecules involved in mediating pain transmission [199]. The upregulation and downregulation of miRNAs have been associated with maladaptive changes in chronic pain at multiple levels, from primary afferents to the DRG, spinal cord, and up to cortical regions [181]. In particular, miRNAs negatively regulate gene expression by canonical binding to their target or directly by interacting with proteins, and by binding to the 3′-untranslated regions (3′ UTR), causing either mRNA degradation or the inhibition of protein translation. For example, a significant downregulation of miR-23a and concomitant increased CXCR4 expression was observed in the spinal glial cells of mice with spinal nerve ligation (SNL)-induced neuropathic pain [200].

Pain sensitization pathways are driven by changes at receptor and channel levels that are responsible for hyperexcitability. Indeed, many studies highlight miRNAs targeting voltage-gated channels or their regulatory subunits that are altered in the neuropathic pain model in rodents [201,202]. For instance, a decrease in the miR-182 level is associated with NaV1.7 upregulation in the nerve injury model [182], and the miR-17-92 cluster seems to be involved in pain perception by downregulating multiple KV channels in the DRG [202]. Also, nerve injury-induced allodynia is correlated with increased expression levels of miR-132-3p in the DRG and dorsal spinal cord [203], while the overexpression of miR-18a, miR-19a, miR-19b, and miR-92a cluster members seem to elicit mechanical allodynia caused by nerve injury [204]. Moreover, the targets of miRNA can be also key signaling factors or pain mediators, such as NGF [205], BDNF [176,206,207], NF-L [208], and AKT3 [209].

### 6.2. Epigenetics of Childhood Trauma: Long-Term Sequelae and Potential for Treatment

Accumulating evidence accentuates the mediating role of epigenetic marks in the molecular mechanisms underlying stressful stimuli in early life, changes in sensitive brain regions, the dysregulation of neuroendocrine signaling, and alterations in immunological pathways [210,211]. Epigenetic changes in childhood trauma events are detected among the pathway implicated in the regulation of the stress response or signaling molecules crucial for brain development, such as BDNF and oxytocin, the hormone that regulates social interactions [8,212,213]. 

Moreover, epigenetic alterations are involved in neurotransmitter signaling, like that of serotonin and dopamine, among the key players in the etiology of psychiatric disorders [214,215]. Studies in animal models support the crucial role of methylation as a modulator of the stress response. For example, offspring experiencing high maternal care in animal models are less likely to develop anxiety-like behavior and display a lowered reactivity of the HPA axis following stressful experiences [216]. Increased levels of DNA methylation of the glucocorticoid receptor gene, *nr3c1*, resulting in a decrease in GR mRNA levels, are observed in the hippocampus and nucleus accumbens of licking–grooming adolescent rats [216,217,218].

Consistently, *nr3c1* promoter hypermethylation is detected in patients who experienced physical, sexual, or emotional abuse in childhood [219,220]. Also, an increase in the methylation rate has been shown at several CpGs of the *nr3c1* gene in a larger cohort of adults with a history of childhood adversity [221]. Human studies have provided further evidence supporting the involvement of miRNA regulation, specifically miR-15a, miR-124, and miR-125b-1-3p, as well as the methylation status of FKBP5, in individuals who have experienced childhood trauma. These findings suggest that these molecular mechanisms play a role in the biological responses to early traumatic experiences in humans [222,223,224]. Interestingly, the Kit Ligand gene (KITLG) hypermethylation is functionally associated with childhood trauma and stress responsiveness in humans [225]. Combinatorial epigenetic regulation is observed for BDNF in animal models as well as in patients; DNA methylation of the BDNF promoter is increased in patients with psychiatric diseases and histories of childhood trauma, suggesting a positive correlation between adverse early-life experience and BDNF hypermethylation [219,226,227,228,229].

Important epigenetic changes also concern the neuropeptide oxytocin known to be implicated in mediating the long-term effects of early adversity on the adult brain [230]. Epigenetic alterations, that is, the region-specific hypermethylation of CpG sites in the oxytocin receptors (OXTR) exist in the hippocampus of rodents [231] and in adult humans who experienced low maternal care [232]. Despite this previous evidence, more recent findings contrast a possible association of OXTR or oxytocin gene methylation with childhood trauma [15,233].

It is important to consider other candidate genes that have demonstrated epigenetic alterations associated with childhood trauma. Among these genes is *Comt*, which encodes the catechol-O-methyltransferase enzyme involved in neurotransmitter metabolism. Additionally, the pro-inflammatory cytokine IL-6 has also been implicated in epigenetic changes related to childhood trauma. These findings highlight the potential impact of childhood trauma on the epigenetic regulation of genes associated with neurotransmission and immune response.

Methylation patterns in the *Comt* gene have been observed in individuals with schizophrenia and depression [234]. Similarly, methylation of IL-6 has been linked to childhood trauma-related phenotypes [235]. These findings highlight the potential role of epigenetic modifications in these genes in the context of mental health and childhood trauma. Furthermore, disruptions in neurotransmitter signaling pathways represent a significant aspect of the neurobiology underlying the long-term effects of early-life trauma. Preclinical studies using a rodent model of low-grooming or low maternal care suggest that epigenetic changes modulate the glutamic, serotoninergic, and dopaminergic systems. The lower level of serotonin and the downregulation of the glutamic acid decarboxylase (GAD1) have been associated with increased levels of DNA methylation and histone 3 acetylation in the hippocampus [188]. Also, the activity of miRNA-135 regulates serotoninergic signaling by targeting the serotonin transporter and receptor [236,237,238].

Also, increased levels of DNA methylation have been detected for the dopamine receptor D1 promoter after maternal separation [239], and miRNA-dependent mechanisms affect dopamine signaling in response to childhood trauma by modulating dopamine receptor expression [240]. Consistently, studies from humans indicate that epigenetic dysregulation of serotoninergic, dopaminergic, and GABAergic signaling is correlated with the long-term sequelae of early-life adversity. DNA methylation patterns of the serotonin transporter gene, as well as specific promoter CpG sites [241,242], or the tryptophan hydroxylase locus (TPH2) involved in serotonin biosynthesis, have been associated with the response to adverse experiences during early life [241]. These epigenetic modifications suggest a potential link between the regulation of serotonin-related genes and the impact of early life adversity. Finally, DNA methylation at the dopamine receptor D2 gene promoter contributes to the epigenetic dysregulation of dopamine [243] and glutamate [244,245] signaling in response to early trauma experience in human studies.

### 6.3. Epigenetics as a New Paradigm for Developing Drugs or Strategies for Pain and Trauma Management

Epigenetics plays a crucial role in the biological response of individuals to stress or adverse experiences by mediating the interaction between genes and the environment. Indeed, all the studies described in this present narrative review shed light on the mechanism exerted by epigenetic alterations in controlling behavior via the translation of environmental stimuli in gene expression regulation. Gaining an understanding of the mechanisms through which injury, inflammation stimulation, or traumatic experiences can induce changes in gene expression is essential for comprehending the underlying causes of biological and behavioral disorders. Such knowledge is crucial and relevant for the development of effective therapeutic strategies aimed at addressing these disorders. Chronic pain patients respond poorly to conventional analgesics. Thus, identifying the molecular determinants of this condition would be essential for developing new mechanism-based therapies. In this context, as mentioned earlier, targeting epigenetic modifications presents a promising avenue for mitigating or preventing the adverse consequences of chronic pain via a more targeted pharmacological approach. By specifically addressing the epigenetic alterations associated with chronic pain, it may be possible to develop interventions that are more precise and effective in managing the condition [194,246]. 

Although much remains to be elucidated, epigenetics is a growing field, especially in research regarding the long-term sequelae of early-life adversity, unraveling how these events alter the health trajectory and indicating possible ways to promote resiliency and moderate potential outcomes in children [247].

Several studies have reported a reversal of epigenetic changes induced by childhood trauma via psychological intervention or anti-depressant administration [15,191,193,211,221,223,235,246,247,248,249]. The strategy of blocking DNMTs via inhibitor administration is the most effective way to prevent DNA hypermethylation. Besides this approach, which shows great promise as a treatment for a pain condition, several side effects on genome stability and mutagenic risk must be considered. In addition, targeting methylation is not a specific strategy and can involve the entire genome [250]. While, currently, several HDAC inhibitors and miRNAs are being investigated in clinical trials for cancer, cardiovascular, neurodegenerative, and inflammatory diseases [15,251,252], the mechanisms and functional specificity of DNA methylation and histones that can be used as potential targets are still unclear. One important limitation is represented by the non-specific activity of HDAC inhibitors across the tissue or brain area and the pleiotropic nature of miRNA targets, which could produce adverse effects. For this reason, it is necessary to identify the unique target gene controlled via specific epigenetic regulators or to deliver epigenetic drugs locally to minimize adverse effects. Considering the many factors, influencing pain perception and modulation, internally and externally, it is important to tailor pain management on the individual level. For this purpose, the integration of omics analysis and big data will be fundamental to characterize and deepen our knowledge of the biological processes and molecular mechanisms involved in pain- and trauma-related disorders in early life, producing a high level of innovation in mental health [8]).

In addition, advanced biotechnology like CRISPR-based epigenetic editing will allow modulating site-specific alterations of DNA in vitro. However, the clinical application of this strategy requires further extensive work and safety evaluation [15,253].

There are important limitations associated with targeting epigenetic changes. Firstly, epigenetic changes in neurogenesis, cognition, synaptic plasticity, and neurodevelopmental and neurodegenerative diseases are known to be dynamic and unstable [15,253]. Secondly, enduring epigenetic marks can exhibit intergenerational or transgenerational heritability, meaning that epigenetic modifications occurring in germline cells can become stable and persist in subsequent generations even in the absence of the original stimuli [223,254]. Tracking inherited epigenetic information and understanding its effects in subsequent generations poses a significant challenge that needs to be addressed [249].

## 7. Final Remarks

In conclusion, despite years of research in patient populations and animal models, the prevalence and impact of early adverse experiences, toxic stress, trauma, and pain continue to increase [12]. To address this issue, there is an urgent need for translational efforts between clinical and preclinical research to identify underlying molecular events, determine neurobiological mechanisms, develop and test novel therapeutic approaches, and translate basic research into safe and effective clinical practices. Modeling human problems, such as pain and trauma, will require using a diverse array of species, as each has unique advantages. While animal models have their advantages in terms of wide possibilities and converging evidence, the complex nature of these human problems often makes it necessary to validate findings in humans. Given the complex multifaceted nature of this relationship [139], in this narrative review, we considered pediatric pain and trauma from different perspectives. As our understanding of these issues improves, we can create more precise preclinical models and ultimately translate our findings back to clinical practice. The goal is to develop effective preventive and therapeutic strategies to improve the lives of those affected by early adverse experiences, toxic stress, trauma, and pain. 

Experiencing pain can be stressful, even traumatizing, especially for young children and adolescents. Though often thought of as separate constructs, there is growing evidence suggesting a complex connection between the two.

While pain and trauma are often thought of as separate constructs, studies have shown that individuals who have experienced trauma are more likely to report pain symptoms, and those with chronic pain are more likely to report symptoms of trauma. This suggests that there may be a shared underlying mechanism or pathway involved in both pain and trauma. Further research is needed to better understand this connection and develop effective treatments that address both pain and trauma symptoms.

## Data Availability

Not applicable.
